# Comparison between subjective and objective evaluations of self-care performance in elderly inpatients

**DOI:** 10.1590/S1679-45082018AO3987

**Published:** 2018-04-19

**Authors:** Delcina Jesus Figueredo, Wilson Jacob-Filho

**Affiliations:** 1 Faculdade de Medicina, Universidade de São Paulo, SP, Brazil.

**Keywords:** Aged, Self care, Efficiency, Caregivers, Idoso, Autocuidado, Eficiência, Cuidadores

## Abstract

**Objetive:**

To identify the functional status in self-care performance of elderly inpatients, through subjective and objective evaluations.

**Methods:**

Fifty-five pairs of elderly and their respective caregivers of both sexes were submitted to subjective (self-rating and rating by caregivers) and objective assessment. The Performance Test of Activities of Daily Living and items in the Instrumental Activities of Daily Living scale were used. Functional status was rated 1 (unable to perform task), 2 (able to perform task with assistance) or 3 (able to perform task unassisted). The agreement rate among different self-rating and rating by caregivers, and objective assessment was calculated by dividing the number of identical responses by the total.

**Results:**

Most elderly patients and caregivers were women (58.2% and 83.6%, respectively). The mean age was 80 years for elderly patients and 58.7 years for caregivers. Low schooling levels (1 to 4 years) prevailed among elderly patients (65.4%), while caregivers often had complete high education (32.7%). Functional status (FN=1, 2 and 3) varied between tasks, and the agreement rate between assessment methods ranged from 58 to 98.1%, particularly in comparisons involving objective assessment.

**Conclusion:**

Self-reported data and data contributed by caregivers must be compared with performance data collected via objective assessment for a reliable appreciation of the true functional status of older adults.

## RESUMO

**Objetivo:**

Identificar o nível de funcionalidade no desempenho dos autocuidados em idosos internados, por meio de avaliações subjetivas e objetivas.

**Métodos:**

Cinquenta e cinco pares de idosos e respectivos cuidadores, de ambos os sexos, submeteram-se a: avaliação subjetiva dos idosos, avaliação subjetiva dos cuidadores e avaliação objetiva do idoso. Foi aplicada a Escala Ecológica de Desempenho de Atividades de Vida Diária, bem como itens das Atividades Instrumentais de Vida Diária. O nível de funcionalidade foi classificado em 1 para “não faz a tarefa”, 2 para “faz com ajuda” e 3 para “faz sem ajuda”. Calculamos o índice de concordância entre as avaliações subjetiva dos idosos, subjetiva dos cuidadores e objetiva do idoso dividindo o número de respostas idênticas pelo total.

**Resultados:**

A maioria dos participantes era do sexo feminino entre os idosos (58,2%) e os cuidadores (83,6%). A média de idade dos idosos foi 80 anos e, dos cuidadores, 58,7 anos. A escolaridade baixa (1 a 4 anos) predominou entre os idosos (65,4%) e a alta (Ensino Superior), entre os cuidadores (32,7%). O nível de funcionalidade (FN=1, 2 e 3), oscilou entre as tarefas avaliadas e o índice de concordância variou de 58 a 98,1%, principalmente nas comparações com a avaliação objetiva.

**Conclusão:**

As informações relatadas por idosos e cuidadores devem ser comparadas às obtidas objetivamente, para melhor identificar a real funcionalidade dos idosos.

## INTRODUCTION

The preservation of functional abilities in older adults is associated with cultural and social values.^(^ ^1^ ^-^ ^3^ ^)^ Self-care is a significant aspect of ageing, as it is thought to be the best healthcare strategy to preserve the ability to perform Activities of Daily Living (ADL) and Instrumental Activities of Daily Living (IADL) in this stage of life.^(^ ^4^ ^-^ ^9^ ^)^ Self-care aims to prevent injuries and conditions leading to hospitalization of older adults, which may reduce ambulatory ability, promote functional decline and affect health dynamics in this population, with significant impacts on the routine of relatives and caregivers.^(^ ^4^ ^-^ ^9^ ^,^ ^10^ ^)^

Caregivers play a pivotal role in bridging the gap between dependent elderly and the health care team. The ability to judge information contributed by both parties enhances the team's capacity to prevent diseases and promote health among ageing adults.^(^ ^11^ ^,^ ^12^ ^)^

Health care delivered to the elderly should be targeted at comorbidities, such as hypertension, diabetes, rheumatologic conditions, heart diseases, dementia, and others. These are indicators of common conditions in this age group, which must be monitored by health care teams so that proper preventive and therapeutic interventions can be implemented.^(^ ^13^ ^-^ ^15^ ^)^ Assessment tools employed by nursing teams lack sensitivity and are often unable to capture valuable information that supports better health planning for older adults.^(^ ^7^ ^,^ ^14^ ^,^ ^15^ ^)^

Subjective and objective assessment strategies can be used to compare reported and observed ability of geriatric patients to perform a given task.^(^ ^7^ ^,^ ^15^ ^)^ Objective assessment provides practical information on the performance of ALD and IADL, thus enabling early detection of disorders and proper planning aimed to overcome functional impairment and promoting self-care.^(^ ^7^ ^,^ ^15^ ^)^

The Performance Test of Activities of Daily Living (PADL) is a sensitive tool for identification of patients suffering from organic or nonorganic disorders, and may be used to measure the progression of hospitalized patients in the short-term. Furthermore, PADL is thought to be one of the best tools for this type of assessment, since it is based on objective and accurate tasks, being the gold-standard test.^(^ ^16^ ^)^ The IADL assessment scale provides data on the ability of older adults to perform certain tasks.^(^ ^7^ ^,^ ^14^ ^,^ ^15^ ^)^ In contrast, objective assessment consists of face-to-face interviews held over the course of hospital stay to investigate self-care performance.

## OBJECTIVE

To identify the functional status in performance of self-care of hospitalized older adults by means of subjective and objective assessments based on the Performance Test of Activities of Daily Living and items in the Instrumental Activities of Daily Living scale.

## METHODS

An observational, cross-sectional cohort study carried out at the geriatric inpatient unit of a quaternary care university hospital, located in the state of São Paulo, from August 2013 through March 2014. Data collection consisted of the applicacation of subjective elderly (ES) and subjective caregivers (SC) and objective evaluation (OB) respectively. The sample included 55 elderly patients-caregiver pairs, regardless of gender. The patients in this study were aged 60 years or over.

However, only geriatric patients were submitted to objective assessment. Assessments were carried out in an individualized manner (*i.e.*, in the absence of the second element of a pair), within two days of hospital admission. In ES and CS, the subjects were asked to rate self-care skills using an assessment scale. In objective assessments, the ability of patients to perform a given simulated task was judged by a rater. The PADL (Performance Test of Activities of Daily Living) scale containing 16 items was used by the author.^(^ ^7^ ^)^

Data collected were stored in Excel spreadsheets and analyzed using the Statistical Package for the Social Sciences (SPSS) for Windows, version 17.0. Patients were quantitatively described using summary measures (mean, median, standard deviation, minimum and maximum value). Absolute and relative frequencies were used to describe qualitative data. Associations between selected characteristics and questionnaires responses were investigated using the χ^2^ test. The level of agreement between subjective and objective assessments was measured using Kappa (K) coefficients, and the agreement index was employed to estimate the percentage of identical responses across different assessments. The study was approved by the Research Ethics Committee, protocol 497.440, CAAE: 15546013. 3.0000.0068.

## RESULTS

Socio-functional characteristics of the 55 pairs studied are shown in table 1. Most geriatric patients and caregivers were women (58.2% and 83.6%, respectively). The mean age of elderly was 80 years, and 58.7 years for caregivers. Most geriatric patients (65.4%) had elementary education, while 32.7% of caregivers had complete higher education. Geriatric patients had 3.15 children on average, compare to 2 children per caregiver. As to kinship, caregivers were the spouse or daughters in 20.7% and 53.4% of cases, respectively.

**Table 1:** Socio-functional characteristics of the study sample in hospitalized patients in the geriatric ward and their caregivers

Variables		Elderly (n=55)		Caregivers (n=55)	
		n (%)	p value	n (%)	p value
Sex					
	Female	32 (58.2)	0.864	46 (83.6)	0.817
	Male	23 (41.8)		9 (16.4)	
Age					
	Mean (SD)	80 (7.24)		58.7 (11.96)	
	Median (minimum; maximum)	80(64:99)		58 (37:84)	
MMSE					
	Mean (SD)	22 (4.99)	0.891	- -	
	Median (minimum; maximum)	23(9:30)		- -	
Katz					
	Mean (SD)	9.42 (3.13)	0.503	- -	
	Median (minimum; maximum)	10 (6:16)		- -	
Lawton					
	Mean (DP)	15.80 (4.56)	0.329	- -	
	Mean (DP)	16 (9:27)		- -	
Children					
	Mean (DP)	3.15 (1.80)		2 (1.64)	
	Mean (DP)	3 (0:6)		2 (0:7)	
Race					
	White	40 (72.7)		38 (69.1)	
	Non-white	14 (25.5)		16 (29.1)	
	Yellow	1 (1.8)		1 (1.8)	
Schooling level					
	Illiterate	7 (12.7)		-	
	Primary education	36 (65.4)		7 (12.7)	
	Secondary education	5 (9.1)		17 (30.9)	
	Secondary education	6 (10.9)		13 (23.6)	
	Complete university education	1 (1.8)		18 (32.7)	
Marital status					
	Married	21 (38.2)		34 (61.8)	
	Single	3(5.5)		8 (14.5)	
	Widower	27 (49.1)		3 (5.5)	
	Divorced	3 (5.5)		7 (12.7)	
	Others	1 (1.8)		3 (5.5)	
Kinship elderly patient and caregiver					
	Wife			12 (20.7)	
	Husband			3 (5.2)	
	Daughter			31 (53.4)	
	Son			7 (12.1)	
	Daughter-in-law/son-of-law			1 (1.7)	
	Grandchild			2 (3.4)	
	Others (friends)			2 (3.4)	

SD: standard deviation; MMSE: Mini-mental State Examination.

**Figure 1:**
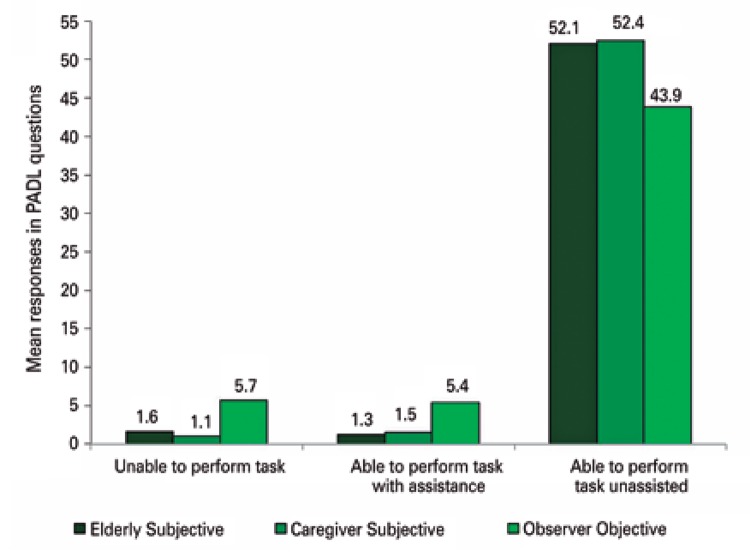
Distribution of mean responses from assessments of elderly subjective, caregiver subjective and objective in the Performance Test of Activities of Daily Living scale

Table 2 shows the distribution of ES, CS and objective assessments of self-care performance scored according to the functional status in tasks listed in the PADL and IADL scales. For example, 27.3% of elderly (ES) and 21.8% of caregivers (CS) reported inability to complete task 4. However, objective assessment revealed that 41.8% were not able to complete that particular task. As for remaining items, the patients were rated unable to perform all three IADL tasks by caregivers, while objective assessment revealed difficulties with some tasks only. Means obtained from different assessment strategies (ES, CS and objective) were compared and graded according to three levels of functional status; similar responses were given by patients and caregivers, while results obtained via objective (rater) assessment differed from both (Figures 1 e 2).

**Table 2:** Subjective assessments of elderly and caregivers and objective assessment about self-care performance in geriatric inpatients

Instrument		ES - FN			CS - FN			OB - FN		
		1	2	3	1	2	3	1	2	3
PADL										
	Drink from a cup	1 (1.8)	-	54 (98.2)	-	-	55 (100)	2 (3.6)	3 (5.5)	50 (90.9)
	Use a tissue to wipe nose	1 (1.8)	-	54 (98.2)	-	-	55 (100)	1 (1.8)	1 (1.8)	53 (96.4)
	Comb hair	1 (1.8)	1 (1.8)	53 (96.4)	1 (1.8)	2 (3.6)	52 (94.5)	5 (9.1)	4 (7.3)	46 (83.6)
	File nails	15 (27.3)	4 (7.3)	36 (65.5)	12 (21.8)	3 (3.6)	40 (76.4)	23 (41.8)	5 (9.1)	27 (49.1)
	Shave/make up	8 (14.5)	2 (3.6)	45 (81.8)	10 (18.2)	4 (7.3)	41 (74.5)	19 (34.5)	5 (9.1)	31 (56.4)
	Lift food onto spoon and to mouth	1 (1.8)	1 (1.8)	53 (96.4)	-	1 (1.8)	54 (98.2)	2 (3.6)	1 (1.8)	52 (94.5)
	Turn faucet on and off	1 (1.8)	2 (3.6)	52 (94.5)	-	2 (3.6)	53 (96.4)	5 (9.1)	6 (10.9)	44 (80)
	Turn light switch on and off	1 (1.8)	1 (1.8)	53 (96.4)	-	1 (1.8)	54 (98.2)	5 (9.1)	7 (12.7)	43 (78.2)
	Put on and remove a jacket	1 (1.8)	4 (7.3)	50 (90.9)	-	4 (7.4)	51 (92.6)	3 (5.5)	5 (9.1)	47 (85.5)
	Put on and remove shoes	1 (1.8)	3 (5.5)	51 (92.7)	-	5 (9.1)	50 (90.9)	3 (5.5)	6 (10.9)	46 (83.6)
	Brush teeth	1 (1.8)	3 (5.5)	51 (92.7)	1 (1.8)	2 (3.6)	52 (94.5)	2 (3.6)	5 (9.1)	48 (87.3)
	Make a phone call	2 (3.6)	8 (14.5)	45 (81.8)	1 (1.8)	12 (21.8)	42 (76.4)	6 (10.9)	17 (30.9)	32 (58.2)
	Sign name	2 (3.6)	2 (3.6)	51 (92.7)	2 (3.6)	3 (5.5)	50 (90.9)	3 (5.5)	4 (7.3)	48 (87.3)
	Turn key in lock	2 (3.6)	1 (1.8)	52 (94.5)	--	6 (10.9)	49 (89.1)	7 (12.7)	6 (10.9)	42 (76.4)
	Tell time	4 (7.3)	-	51 (92.7)	2 (3.6)	4 (7.3)	49 (89.1)	4 (7.3)	7 (12.7)	44 (80)
	Stand up and walk a few steps, and sit back down	1 (1.8)	-	54 (98.2)	-	1 (1.8)	54 (98.2)	1 (1.8)	5 (9.1)	49 (89.1)
IADL scale items										
	Medications: able to store, recognize and take them	1 (1.8)	11 (20)	43 (78.2)	-	18 (32.7)	37 (67.3)	2 (3.6)	18 (32.7)	35 (63.6)
	Handling money	1 (1.8)	6 (10.9)	48 (87.3)	-	13 (23.6)	42 (76.4)	2 (3.6)	11 (20)	42 (76.4)
	Shopping	1 (1.8)	9 (16.4)	45 (81.8)	1 (1.8)	17 (30.9)	37 (67.3)	3 (5.5)	13 (23.6)	39 (70.9)

FN: 1 - unable to perform task; 2 - able to perform task with assistance; 3 - able to perform task unassisted. K: Kappa; Agreement rate (%); McNemar test. (-) Caselas test with data equal to zero. PADL: Performance Test of Activities of Daily Living; FN: Functional level and IADL: Instrumental Activities of Daily Living; ES: subjective of elderly; SC: subjective of caregivers; OB: objective.

**Figure 2:**
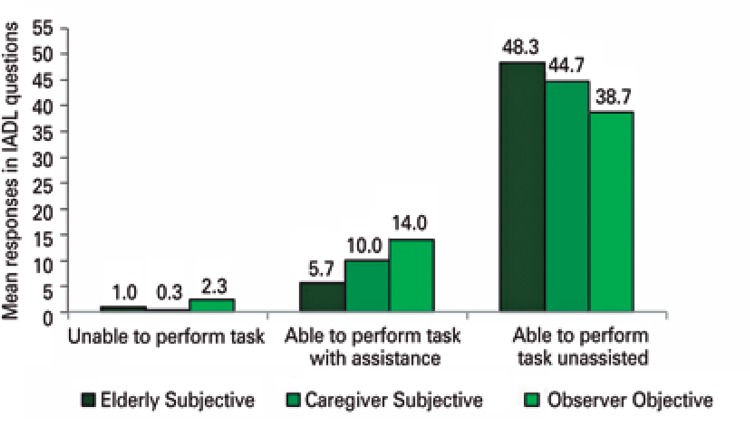
Distribution of mean responses from assessments of elderly subjective, caregiver subjective and objective in Instrumental Activities of Daily Living items

**Figure 3:**
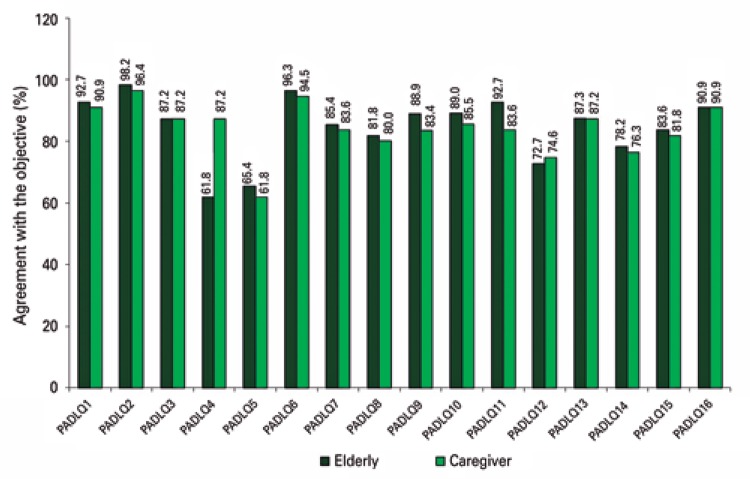
Distribution of agreement between patient and caregiver data and caregivers as compared to objective assessments in Performance Test of Activities of Daily Living scale

**Figure 4:**
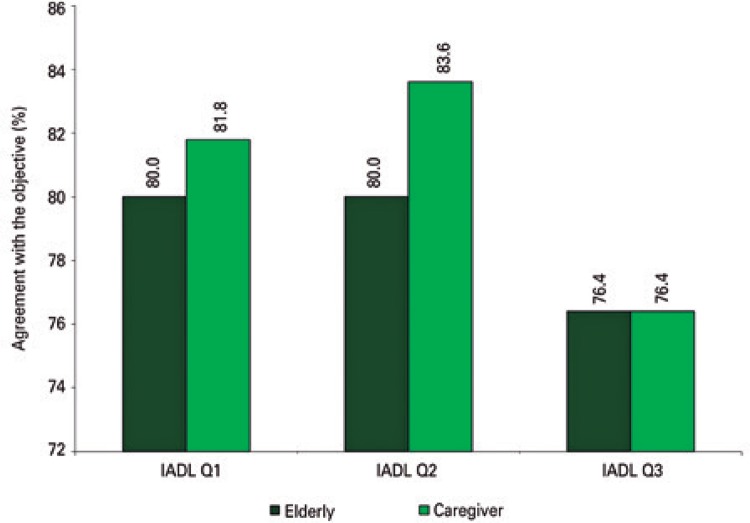
Distribution of agreement between patient and caregiver data as compared to objective assessments in Instrumental Activities of Daily Living scale

Comparative analysis of data derived from ES and objective assessments are displayed in table 3. The levels of agreement were reasonable (tasks 1, 3, 4, 5, 7, 8, 13, 15 and 16; 02.0≥K≤0.39), moderate (tasks 6, 9, 10 and 12; 0.40≥K≤0.59) or substantial (task 2; 0.60≥K≤0.79). Comparisons between CS and objective assessment of performance in the same tasks revealed reasonable (tasks 3, 4, 5, 6, 7, 10, 13, 14, 15 and 16; 0.20≥K≤0.39) or moderate (task 12; 0.40≥K≤0.59) correlation; the p-value was non-significant and the index of agreement ranged from 58.0 to 96.4%. In ES *versus* CS comparisons, correlation was regular (tasks 4, 7, 11 and 14; 0.20≥K≤0.39), moderate (tasks 10, 12, 13 and 15; 0.40≥K≤0.59) or substantial (tasks 3 and 6; 0.60≥K≤0.79); the p-value was also non-significant and the index of agreement ranged from 65.4 to 98.1%. Overall, performance in the above-mentioned tasks differed between ES, CS and objective assessment. Despite reported ability to perform these tasks, the patients showed difficulties when it came to actually performing these same tasks. As regards remaining items in the IADL scale, ES and objective and ES and SC comparisons produced Kappa coefficients of (0.20≥K≤0.39) for task one (1) and (0.40≥K≤0.59) for tasks 2 and 3. The p-value remained non-significant and the index of agreement ranged from 58 to 96.4%. The level of agreement between different assessment strategies (ES, CS and objective) across the various tasks can be appreciated in figures 3 and 4.

**Table 3:** Subjective assessments of elderly and caregivers compared to objective assessment about self-care performance in Performance Test of Activities of Daily Living tasks and items of Instrumental Activities of Daily Living in inpatients

Instrument		Performance assessment								
		ES *versus* OB			CS *versus* OB			ES *versus* CS		
		Kappa	p value	Agreement rate (%)	Kappa	p value	Agreement rate (%)	Kappa	p value	Agreement rate (%)
PADL										
	1. Drink from a cup	0.316	0.027	93.0	-	-	91.0	-	-	98.1
	2. Use a tissue to wipe nose	0.661	0.007	98.2	-	-	96.4	-	-	98.1
	3. Com hair	0.330	0.033	87.2	0.378	0.011	87.0	0.784	0.002	98.1
	4. File nails	0.315	0.039	62.0	0.378	0.033	58.0	0.248	0.121	65.4
	5. Shave/make up	0.287	0.009	65.4	0.252	0.168	62.0	0.043	0.772	65.4
	6. Lift food onto spoon and to mouth	0.580	0.002	96.3	0.233	0.027	94.5	0.661	0.007	98.1
	7. Turn faucet on and off	0.388	0.006	85.4	0.271	0.008	84.0	0.373	0.104	94.5
	8. Turn light switch on and off	0.249	0.056	82.0	0.130	0.119	80.0	-0.04	0.963	94.5
	9. Put on and remove a jacket	0.494	0.004	87.0	0.187	0.198	82.0	0.165	0.478	87.2
	10. Put on and remove shoes	0.496	0.001	89.0	0.370	0.013	85.4	0.403	0.032	90.0
	11. Brush teeth	0.606	<0.001	93.0	0.041	0.294	84.0	0.252	0.457	90.0
	12. Make a phone call	0.425	<0.001	73.0	0.477	<0.001	74.5	0.417	0.003	80.0
	13. Sign name	0.318	0.009	87.2	0.361	0.022	87.2	0.526	0.001	93.0
	14. Turn key in lock	0.197	0.032	78.1	0.229	0.001	76.3	0.301	0.031	89.0
	15. Tell time	0.352	0.001	84.0	0.339	0.002	82.0	0.469	0.001	90.0
	16. Stand up and walk a few steps, and sit back down	0.270	0.007	91.0	0.263	0.082	91.0	-0.010	0.847	96.3
IADL items										
	1. Medications: able to store, recognize and take them	0.542	<0.001	80.0	0.609	<0.001	82.0	0.510	<0.001	80.0
	2. Handling money	0.356	0.003	80.0	0.556	<0.001	84.0	0.232	0.064	76.3
	3. Shopping	0.380	0.009	76.4	0.474	<0.001	76.3	0.360	0.006	74.5

McNemar test. (-) Caselas test with data equal to zero.

PADL: Performance Test of Activities of Daily Living; IADL: Instrumental Activities of Daily Living.

## DISCUSSION

Objective assessment of performance in daily living activities was not consistent with subjective reports provided by patients or respective caregivers. This gave us some insight into the extent to which elderly patients are able to accurately rate their own ability to perform essential, as well as and more complex, instrumental activities of daily living. Objective assessment is thought to be the gold-standard to assess ability of the elderly when performing these activities, allowing better classification of functional status, which favors the implementation of actions aimed to address the specific needs of this population.

The sociodemographic and functional profiles of the 55 elderly-caregiver pairs in this sample reflected those reported in other studies.^(^ ^17^ ^-^ ^19^ ^)^ Prevailing low levels of education among elderly patients contrast with the higher levels of education among caregivers (*e.g.*, complete higher education), as reported in literature.^(^ ^17^ ^-^ ^19^ ^)^

Some data used in this study, such as the systematic evaluation Mini-Mental State Examination (MMSE), were extracted from the database derived from the Comprehensive Geriatric Assessment (CGA),^(^ ^20^ ^)^ carried out in the geriatric inpatient unit prior to admission of the elderly. Mini-Mental State Examination data were used to score elderly patients in this sample according to cognitive function; the mean score was 22 points, compared with 21.4 points reported in literature. Activities of Daily Living and IADL scale means (9.42 and 15.80 points, respectively) were similar to those reported in previous studies,^(^ ^17^ ^-^ ^21^ ^)^ indicating that most elderly patients in this sample had preserved functional status.

Comparisons between objective, ES and CS assessments revealed reasonable agreement between responses, with reasonable Kappa coefficients for most PADL tasks and a non-significant p-value. The agreement rate pointed to greater mismatch between responses in some tasks. When ES and CS were compared, the elderly rated themselves higher than their caregivers did. Similar data have been reported in literature in most cases, showing that caregivers tend to underestimate the functional abilities of geriatric patients.^(^ ^22^ ^)^ A different study reported substantial kappa values for PADL, confirming that caregivers must be more aware of the functional and health status of their patients.^(^ ^23^ ^)^ Agreement between ES and CS data regarding PADL performance has also been reported.^(^ ^24^ ^)^

Kappa values associated with items in the IADL scale were either reasonable (tasks 2 and 3) or moderate (task 1). In one study comparing ES and CS, Kappa values were moderate (“managing money”) or equal to zero (“managing medication” and “shopping”).^(^ ^22^ ^)^ In contrast, the index of agreement varied between “shopping” and “managing money”.^(^ ^24^ ^)^

Self-care performance of PADL tasks 1 to 16 was associated with reasonable to substantial Kappa values. The agreement rate revealed discrepancies between self-rated and objectively assessed skills. These results show that self-rated performance differed significantly from performance *in loco* under the supervision of a rater, as previously reported.^(^ ^7^ ^)^

Subjective information given by caregivers was not consistent with self-reported data, since geriatric patients tended to rate themselves higher than their caregivers did. Similar differences have been described in literature.^(^ ^17^ ^)^

Comparisons between CS and objective assessment of self-care skills yielded reasonable kappa values for some PADL tasks (1 to 16) and items in the IADL scale. Despite reported ability by caregivers, patients were unable to perform the tasks during objective assessment in some cases. Instrumental Activities of Daily Living scale items 1 to 3 were associated with moderate or substantial kappa values, and the agreement rate revealed slight inconsistency with the objective assessment of task 3. The comparative analysis of ES, CS and objective assessments showed that elderly patients overestimated their functional status as compared to caregivers, while objective rating was comparatively poorer. There is a need to extend public policies targeted at older adults and respective caregivers, so as to provide them with care and self-care guidance. Also, further studies are warranted to detect and develop better self-care strategies for older adults.

## CONCLUSION

This study emphasized the importance of approaches comparing self-reported data with data provided by caregivers, or collected via objective assessment for improved understanding of self-care skills in geriatric patients. Data contributed by caregivers are important, given they are in charge of elderly patients and, therefore, in a position to observe gradual changes taking place over the ageing process course.

Agreement between data contributed by patients and respective caregivers, and those collected via objective assessment, was poor. This fact should be taken into account in care planning in hospitals or nursing homes, since geriatric patients and respective caregivers tend to overestimate true self-care skills, with potential safety and health risks for patients. Therefore, systematic, objective assessment of major feeding and hygiene tasks is recommended to ensure these will be performed safely and effectively.
